# The Silent Conquest: The Journey of Micro- and Nanoplastics Through Children’s Organs

**DOI:** 10.3390/toxics13100812

**Published:** 2025-09-24

**Authors:** Elena Esposito, Francesco Fabrizio Comisi, Vassilios Fanos, Antonio Ragusa

**Affiliations:** 1 Neonatal Intensive Care Unit, Department of Surgical Sciences, AOU Cagliari, University of Cagliari, 09124 Cagliari, Italy; elena.esposito30@gmail.com (E.E.); vafanos@tiscali.it (V.F.); 2 Obstetrics and Gynecology Unit, Sassuolo Hospital, 41049 Sassuolo, Italy; antonio.ragusa@gmail.com

**Keywords:** microplastics, nanoplastics, micro (nano) plastics, the first 1000 days, environmental exposure, early-life vulnerability, biomonitoring, child health

## Abstract

Micro- and nanoplastics (MNPs) are emerging environmental contaminants with increasing evidence of bioaccumulation in human tissues and potential toxicological effects. While extensive studies in the literature have investigated MNP exposure and health risks in adult populations, data specific to pediatric age remain scarce and fragmented. This narrative review represents the first integrated synthesis of current evidence on MNP exposure during early life, including the critical period of the first 1000 days, examining routes of absorption (oral, inhalational, dermal, and iatrogenic), biological distribution, and organ-specific effects in infants and children. Special attention is given to the presence of MNPs in pediatric lungs, thyroid, and intestinal microbiota, as well as to emerging non-invasive biomarkers for exposure assessment. The developing physiology of children, characterized by immature detoxification systems, critical windows of vulnerability, and prolonged life-course exposure, amplifies concern for long-term health consequences, including endocrine disruption, immune dysregulation, and neurodevelopmental impairment. This work fills a critical knowledge gap by consolidating pediatric data into a single comprehensive resource, and it is intended to serve both as a reference point for clinicians and researchers and a catalyst for future studies aimed at safeguarding child health in an increasingly plastic-contaminated world.

## 1. Introduction

Over the past few years, a sharp uptick in scientific investigations has underscored that plastics, although ubiquitous in daily life for their practical advantages, can inflict significant environmental harm [1]. Among these materials, microplastics (MPs), defined as particles smaller than 5 mm, have received particular attention. MPs are typically classified into two categories [2]: primary MPs, produced intentionally as industrial pellets and incorporated into personal care products [3], and secondary MPs, originating from plastic waste that progressively fragments through photo- and thermo-oxidative reactions and mechanical abrasion [4]. If the particles are smaller than 100 nm, they are termed nanoplastics [5]. MPs can be further characterized by their polymer composition and morphology traits tightly linked to the source product from which they originate [6]; these include polyethylene (PE), polypropylene (PP), polystyrene (PS), polyvinyl chloride (PVC), polyethylene terephthalate (PET), polycarbonate (PC), polymethacrylate (PMMA), and polyurethane (PU) [7]. However, polyethylene, polypropylene, and polystyrene are the three most encountered polymers [8], occurring in several household and personal care products [9,10]. The morphology of MPs is likewise diverse, encompassing fibers, microbeads, fragments, nurdles, and expanded polystyrene [7]. MPs are rarely chemically pure: they are often associated with a heterogeneous mixture of organic molecules and/or metals. Commercial plastics contain additives that can leach from the polymer matrix into surrounding environments or tissues because they are not covalently bound. Furthermore, the hydrophobic surface of MPs can adsorb environmental contaminants, particularly polyaromatic hydrocarbons [11]. At the population scale, in the European Union alone, annual environmental emissions are estimated at 75,000–300,000 tons of MPs [12]. Microplastics have been detected in marine waters [13], terrestrial soils [14], the atmosphere [15], and tap water [16], in both densely populated areas and remote regions far from human settlements [17]. Recent studies indicate that MPs can enter the human body and have been identified in multiple organs and tissues [18]. Multiple studies have quantified microplastic contamination in bottled water. Mason et al. [19] reported an average of ~325 microplastic particles per liter (>6.5 µm), with 93% of bottles contaminated across 11 global brands. Using advanced microscopy, Qian et al. [20] detected approximately 240,000 particles per liter (including nanoplastics <1 µm), with 90% of particles falling in the nanoplastic range [21]. The study by Leslie et al. in 2022 was the first to reveal and quantify plastic polymers in human blood [22]. MPs have also been shown to cross the placental barrier [23], which is already known to be permeable to various potentially toxic substances [24]. Some studies have documented the presence of MPs in critical biological systems such as the central nervous system, highlighting their capacity to reach and accumulate in protected or functionally essential compartments [25]. Beyond environmental concerns, microplastic exposure may exert direct human effects [5]; in this regard, the Developmental Origins of Health and Disease framework posits that epigenetic alterations during fetal life may predispose individuals to adult-onset diseases [26,27]. This perspective reinforces concerns that exposure to microplastics is particularly problematic in infants and children because their metabolic pathways differ from those in adults; moreover, the amount of exposure across their life cycle is expected to increase further for each passing generation [5]. In addition, children and adolescents experience age-dependent windows of susceptibility and chemical sensitivities distinct from adults [28,29,30]. Building on these concerns, particularly the heightened vulnerability of developing organs, this review aims to synthesize evidence of MNP presence and biodistribution in organs in the pediatric age; appraise exposure pathways pertinent to children; evaluate detection methods and plausible mechanisms of harm; and delineate priority knowledge gaps and their clinical/public health implications (Table 1).

## 2. Methods

This article was conceived as a narrative review aiming to provide an updated and integrated synthesis of the current knowledge on MNP exposure during early life and its potential implications for pediatric health. The methodological approach followed international standards for narrative reviews in environmental and pediatric health research.

### 2.1. Literature Search Strategy

A comprehensive literature search was conducted using PubMed, Scopus, Web of Science, and Google Scholar, encompassing studies published between August 2009 and June 2025. Keywords and MeSH terms used in combination included: “microplastics”, “nanoplastics”, “plastic particles”, “pediatrics”, “infants”, “children”, “developmental exposure”, “endocrine disruption”, “neurodevelopment”, “toxicology”, “biomonitoring”, and “environmental pollution”. Boolean operators (“AND”, “OR”) were used to refine and broaden the search as needed. Across the included studies, micro- and nanoplastics were typically identified using Fourier-transform infrared (FTIR) spectroscopy, Raman microscopy, or pyrolysis–gas chromatography/mass spectrometry (Py-GC/MS), which remain the most widely applied techniques in environmental and human biomonitoring.

### 2.2. Inclusion Criteria

Given the narrative nature of this review, we considered a broad range of peer-reviewed publications written in English. Priority was given to studies addressing MNP exposure during early life, including the prenatal, neonatal, and pediatric periods. Relevant contributions included both human and experimental studies, with a particular focus on works investigating exposure pathways, biological matrices, potential health effects, or mechanisms of toxicity. In addition to original research articles, we also included reviews, case reports, and technical or policy documents issued by international agencies or expert working groups. Publications lacking any relevance to child health were not considered in the core analysis.

### 2.3. Exclusion Criteria

Studies were excluded if they were not published in English, if they lacked peer-reviewed status, or if they provided insufficient methodological details to allow meaningful interpretation. Reports that did not address early-life or pediatric health contexts, or that focused exclusively on adult populations without relevance to developmental or pediatric outcomes, were also excluded.

### 2.4. Study Selection and Data Extraction

Titles and abstracts were screened independently by two authors (E.E. and F.F.C.). Full texts of potentially relevant studies were retrieved and reviewed for inclusion. Data extracted included: type of study, population or matrix analyzed, detection methods, type and concentration of MNPs, health outcomes assessed, and key findings. Studies were grouped into thematic categories covering routes of exposure, biological detection, organ-specific effects, toxicological and epigenetic mechanisms, clinical implications, and preventive strategies.

### 2.5. Critical Appraisal and Synthesis

Included studies were evaluated for methodological quality, relevance to the pediatric setting, and level of evidence using adapted principles from the GRADE framework. Findings were synthesized narratively, with a focus on integrating data across different disciplines, including toxicology, endocrinology, developmental biology, and environmental science. Particular attention was given to studies published from 2019 onwards, in view of the rapidly evolving nature of this research field.

## 3. Exposure Pathways and Sources of MNPs in Children

**Figure 1:**
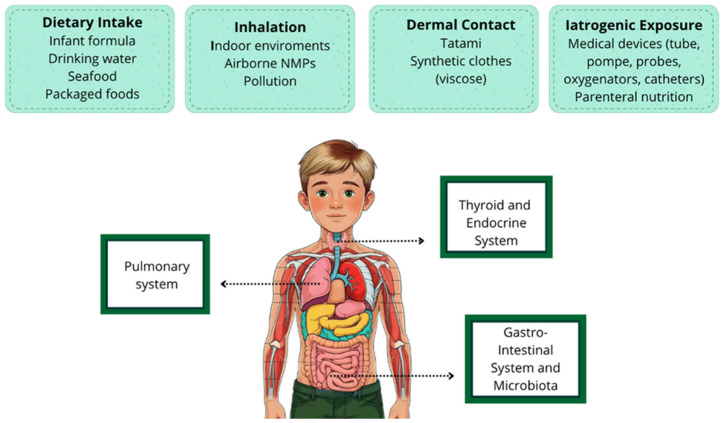
Main routes of entry of MNPs into the human body in children. The figure also illustrates some of the most important organs and systems involved in pediatric age.

Children are particularly susceptible to micro- and nanoplastic exposure due to their unique physiology, behaviors, and developmental stages. The main sources of exposure to MNPs in pediatric age are illustrated in Figure 1. It is important to highlight that, in this regard, available studies are still limited.

### 3.1. Dietary Intake

Infant formula, often the sole nutritional source for infants in early life, has been identified as a significant source of microplastic contamination. In a study conducted in Poland, microplastics (MPs) were detected in all analyzed formula samples, with an average of 42 ± 27 particles per 100 g. The most frequently identified polymers included polyamide (PA), PE, PP, and PET, appearing as fibers, fragments, and films. The estimated daily intake (EDI) for infants aged 0–6 months fed exclusively on these formulas was calculated at 49 ± 32 MPs/day [31]. A Chinese study reported lower MP levels in formulas but emphasized the role of polypropylene feeding bottles as a source of contamination during formula preparation. Heating significantly increased MP release, highlighting that both the formula and the preparation method contribute to exposure [32]. Feeding bottles subjected to high-temperature steam sterilization can release up to 16.2 million MPs per liter into baby formula. This contamination is linked to the degradation of materials such as polypropylene, polycarbonate, and polyphenylene sulfone [33]. Additionally, the mechanical action of opening and closing bottles can release between 53 and 393 particles per milliliter over 100 cycles, potentially exposing infants to 16.3 to 117.3 MPs per feeding, along with other compounds like calcium stearate and silicone [34]. Dairy-based products, including skimmed, whole, powdered milk, and infant formulas, are also susceptible to MP and MNP contamination through migration from plastic packaging. These particles may interact with macronutrients and interfere with digestion and nutrient absorption [35].

Seafood is another possible source of MP exposure. Alongside microplastics, seafood may contain heavy metals, persistent organic pollutants, and pesticides, all of which pose potential risks to child development [36].

### 3.2. Inhalation

Microplastics are prevalent in indoor environments, where children spend most of their time, often in close contact with the floor. A study conducted in kindergartens showed that materials like tatami mats and carpets release airborne MPs. The number of MPs on children’s hands increased from 1.85 ± 1.39 to 4.2 ± 3.05 after a day at kindergarten (*p* < 0.0001), suggesting a high level of exposure in these settings [37]. Indoor air also contains microfibers shed from synthetic clothing, carpets, and upholstered furniture. These particles can be inhaled and may cause respiratory irritation or inflammation, particularly in children with higher respiratory rates and ongoing lung development [38].

### 3.3. Dermal Contact

Children frequently touch surfaces and place their hands in their mouths, increasing the relevance of dermal contact as an exposure route. The same kindergarten study found a direct link between environmental MPs and their presence on children’s hands and in saliva, suggesting a transfer through skin contact followed by ingestion [39]. The widespread use of synthetic fabrics in children’s clothing, especially materials like viscose, further contributes to MP exposure. These fabrics are increasingly replacing natural fibers, raising concerns about skin-level contact with plastic particles [40]. Current evidence suggests that dermal exposure in children mostly reflects external contamination rather than true systemic absorption, although compromised skin barriers (e.g., in preterm neonates or atopic dermatitis) may increase uptake. A pioneering study conducted in three residential complexes in Kielce, Poland, found microplastics in all 27 sand samples collected from nine urban sandboxes, with concentrations ranging from 60 to 5540 particles per kilogram of sand, highlighting the potential for dermal exposure in children through direct contact with contaminated surfaces [41].

Comparisons between microplastic compositions inside and outside playgrounds indicate that plastic structures and materials used in these areas significantly contribute to higher contamination levels, suggesting that dermal exposure to microplastics is more frequent in parks with plastic-based playground equipment [42].

### 3.4. Iatrogenic Exposure

Medical devices used in pediatric care can be a direct source of MNP exposure. A study on children undergoing congenital heart surgery with cardiopulmonary bypass (CPB) showed that plastic components in the CPB circuit (tubing, pumps, oxygenators) released a mix of polymers, including PS, PE, PP, PVC, and PA6. MNP levels in the blood increased significantly after CPB and were associated with longer bypass time, higher white blood cell counts, and elevated neutrophil levels, suggesting an inflammatory response [43,44]. In neonatal and pediatric intensive care units, microplastics have also been found to migrate from infusion circuits into parenteral nutrition solutions. An ex vivo study showed that both crystalloid fluids and lipid emulsions contain MPs, mainly PET and PP, ranging from 25 to 175 µm. Larger particles were more abundant at the beginning of infusion, indicating possible leaching from the plastic components [45]. A clinical study in 36 preterm infants in a Neonatal Intensive Care Unit (NICU) revealed significant exposure to di(2-ethylhexyl) phthalate (DEHP), a common plasticizer. Metabolites like mono(2-ethyl-5-hydroxyhexyl) phthalate (MEHHP) were found in over 80% of urine samples, especially during the first four weeks of life. Infants under 1000 g showed higher levels, particularly those requiring prolonged use of medical devices such as catheters, endotracheal tubes, and feeding lines [46].

## 4. Organ-Specific Effects in Children

While several studies have explored the adverse health effects of micro- and nanoplastics in the general population, a significant research gap persists regarding their specific impact on children. Pediatric populations are particularly vulnerable to environmental pollutants due to critical developmental windows and immature physiological defense mechanisms. A comprehensive review on microplastics in the human body of adults confirmed the distribution of MNPs across various organs and body fluids, highlighting significant variability in particle counts, also depending on the individual’s health status. For example, fecal samples from patients with intestinal diseases contained higher numbers of MPs compared to healthy controls. Table 2 summarizes microplastic concentrations identified in adult human tissues and organs, highlighting internal exposure levels [45,47,48,49].

Current estimates suggest total body accumulation ranges from 14,230 to 17,091 particles, corresponding to 37.26–46.53 mg, with the lungs identified as the primary deposition site. Differences in biological membrane permeability, overall health status, and exposure routes, particularly ingestion and inhalation, are believed to account for these variations. Notably, individuals with inflammatory bowel disease exhibit significantly higher MP levels in fecal samples. Moreover, transplacental passage of MPs has been documented, underscoring fetal susceptibility to microplastic exposure during early development [46]. A growing body of experimental literature also suggests that MPs may exert carcinogenic effects, as demonstrated in both in vitro and in vivo models [50]. The presence of micro- and nanoplastics in the brain falls outside the scope of this review, as it has already been thoroughly addressed in prior publications [25]. Rather, this review explores the current understanding of MNP detection in pediatric populations, with a specific focus on their presence in the lungs, thyroid gland, and gut microbiota.

### 4.1. Pulmonary System

Recent findings have documented the presence of MPs in the lower respiratory tract of children, indicating that inhalation constitutes a major route of pediatric exposure. In a study analyzing bronchoalveolar lavage fluid (BALF) from children with respiratory diseases, 89.6% of samples contained MPs, with a mean concentration of 4.31 particles per 10 mL [36]. The most prevalent polymers were PP (41.9%) and PE (19.4%), lightweight plastics commonly encountered in domestic settings. These particles, typically <20 µm in diameter, are capable of reaching the distal airways and alveoli. Young children may be particularly exposed due to age-specific behaviors (e.g., crawling, hand-to-mouth activity) and prolonged contact with indoor dust. Additionally, their underdeveloped respiratory and immune systems may be less efficient in clearing inhaled particulates. The study reported an inverse correlation between age and MPs burden in BALF, with higher concentrations observed in younger children. Urban residence was also associated with significantly greater pulmonary MP levels compared to rural environments. Although no statistically significant differences were detected between children with asthma and those with community-acquired pneumonia (CAP), markedly elevated MPs concentrations were observed in severe CAP cases. These findings suggest that MP accumulation may exacerbate pre-existing pulmonary inflammation by impairing key defense mechanisms such as alveolar macrophage phagocytosis. Collectively, this evidence emphasizes the need for targeted research into the role of MPs in pediatric respiratory pathology and the development of effective preventive interventions [36].

### 4.2. Thyroid and Endocrine System

**Figure 2:**
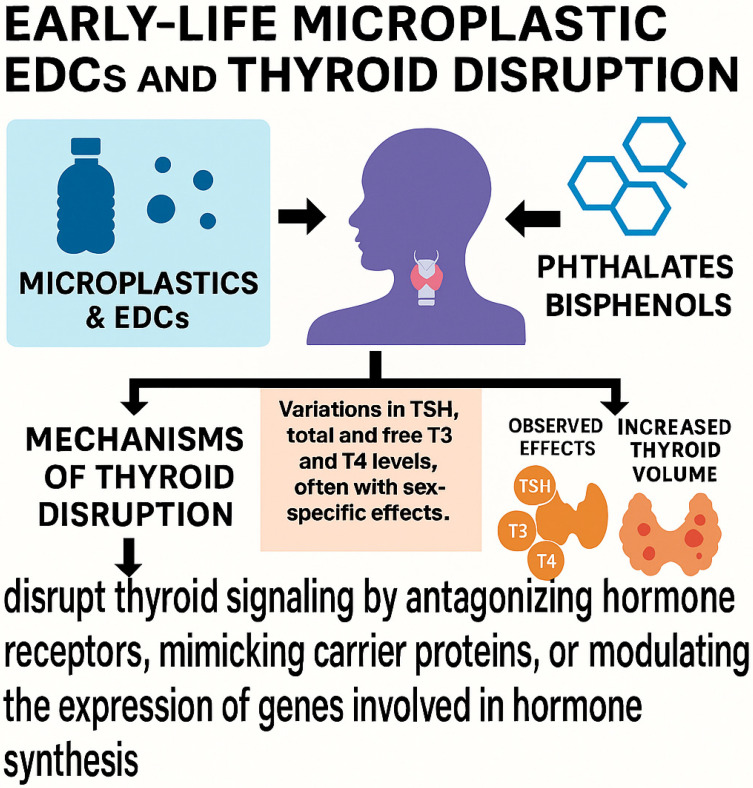
How MNPs and plastic derivatives alter thyroid gland function in childhood.

Microplastics and their associated endocrine-disrupting chemicals (EDCs), including phthalates and bisphenols, have been shown to interfere with thyroid function during early life stages [51]. Given the thyroid’s key role in neurodevelopment, growth, and metabolic regulation, children are especially sensitive to such disruptions. It remains challenging to distinguish direct particle-mediated effects on the thyroid from those mediated by plastic-associated chemicals such as bisphenols and phthalates, which may act through receptor binding or epigenetic modulation. Phthalates, ubiquitous in consumer plastic products, have been linked to alterations in thyroid hormone levels. Prenatal exposure to DEHP, for instance, has been associated with changes in TSH and free T4 levels in neonates. A 2024 systematic review confirmed that multiple phthalate metabolites significantly correlate with altered thyroid hormone profiles in children and adolescents, raising concern for subclinical thyroid dysfunction [52]. Bisphenols (BPs), notably BPA and its analogues (BPS, BPAF), have also been implicated. These compounds have been associated with variations in TSH, total and free T3 and T4 levels, often with sex-specific effects. For example, prenatal BPA exposure has been linked to increased TSH levels in neonates, whereas BPS exposure has been associated with decreased TSH in males. Mechanistically, bisphenols may disrupt thyroid signaling by antagonizing hormone receptors, mimicking carrier proteins, or modulating the expression of genes involved in hormone synthesis. In a cross-sectional study involving Chinese children aged 9 to 11 years, BPA exposure was associated with increased thyroid volume and a higher prevalence of thyroid nodules with advancing age. Importantly, these disruptions may not exhibit a simple dose–response relationship; even low-level exposures during sensitive developmental periods can exert significant endocrine effects [53]. Taken together, these findings highlight the potential for early-life microplastic exposure to result in long-term thyroid and neuroendocrine dysfunction. Figure 2 illustrates the main effects of MNPs on the thyroid during childhood.

### 4.3. Gastrointestinal System and Microbiota

Ingestion of MPs has been associated with significant alterations in the gut microbiota of children. A 2022 cross-sectional study in Xiamen, China, examined 69 stool samples from children aged 3 to 6 years using pyrolysis-gas chromatography-mass spectrometry (Py-GC/MS) to quantify MPs and characterize microbial diversity [54]. Four primary polymer types, PVC, PET, PE, and PA6, were detected in 85.5% of the samples. Higher concentrations of PVC and PE were significantly correlated with reduced alpha diversity of the gut microbiota. Additionally, MP exposure was inversely associated with the abundance of beneficial microbial genera, including Alistipes and Parabacteroides, which are known for their anti-inflammatory and gut-protective functions. Conversely, elevated PVC levels were positively correlated with *Faecalibacterium* spp. Abundance, a genus with a complex and still poorly understood role in pediatric inflammatory bowel disease. Dietary and behavioral factors also influenced MP levels. The use of silicone feeding bottles and high dairy intake were associated with increased stool MP concentrations. Children who required longer meal durations exhibited higher PVC levels, likely due to prolonged exposure of food to airborne MPs. These findings provide compelling evidence that MPs are not only ingested and excreted by children but also capable of disrupting the intestinal microbial ecosystem. Such dysbiosis may predispose to immune dysregulation, metabolic disturbances, and increased susceptibility to chronic inflammatory diseases later in life. Beyond compositional shifts, microplastics may perturb the balance between resident and transient microbial communities, disrupting ecological homeostasis and impairing gut barrier integrity. These alterations could facilitate systemic translocation of luminal antigens and plastic-associated chemicals, amplifying inflammatory and metabolic consequences [54].

## 5. Pathogenic Mechanisms

Microplastics may adversely impact pediatric health through multiple pathogenic mechanisms. Due to their microscopic size, MPs can penetrate biological tissues and cross physiological barriers, including the placental, cutaneous, intestinal, and blood–brain barriers, triggering local and systemic inflammatory responses and oxidative stress. Size-dependent mechanisms are critical, as nanoplastics are more likely than microplastics to cross epithelial barriers, enter systemic circulation, and accumulate in sensitive organs. Toxicokinetics appear highly size-dependent, with smaller particles showing higher translocation efficiency and broader tissue distribution, whereas larger MPs are more likely to remain confined to the gastrointestinal tract. In neonates, particularly preterm infants, the skin barrier is structurally immature and more permeable. Additionally, pediatric skin affected by conditions such as atopic dermatitis demonstrates increased permeability, facilitating the transdermal absorption of MNPs [55]. From a chemical standpoint, MPs can leach toxic additives such as phthalates, BPA, and flame retardants, which are known to disrupt endocrine and neurodevelopmental processes. In pediatric settings, risk may derive both from additives inherently present in plastics (DEHP, BPA) and from pollutants adsorbed on particle surfaces (PAHs, heavy metals), with relative contribution likely depending on exposure context. Moreover, MPs can adsorb environmental pollutants, including heavy metals, pesticides, and persistent organic compounds, thereby enhancing their bioavailability and toxicity upon ingestion. Notably, EDCs are not covalently bound to plastic polymers, enabling their release into systemic circulation, breast milk, and other biological fluids, with subsequent accumulation in endocrine tissues [56]. MPs also interact with heavy metals through mechanisms such as electrostatic attraction, complexation, and electron exchange. Environmental aging processes, including UV exposure, oxidation, and thermal degradation, modify the surface properties of MPs, increasing porosity, surface charge, and metal-binding capacity, thereby amplifying their environmental and biological reactivity [57]. Another key mechanism involves gut dysbiosis: microplastic exposure has been shown to alter intestinal microbiota composition, impairing immune regulation, nutrient absorption, and neurodevelopmental signalling via the gut–brain axis. These changes have been linked to an increased risk of gastrointestinal disorders and may have long-term implications for child health [54]. Inhaled MPs can be phagocytosed by alveolar macrophages [36], damaging the pulmonary barrier and, in some cases, entering systemic circulation. This may facilitate systemic inflammatory responses and expose distant organs, including the central nervous system, to plastic-related toxicants. The convergence of these mechanisms may contribute to neurodevelopmental and behavioral disorders in children, underscoring the importance of minimizing early-life exposure to microplastics as a public health priority [58].

## 6. Exposure Biomarkers

Biomonitoring of microplastic exposure in children relies on the detection of MPs in various biological matrices that serve as biomarkers of exposure.

Blood was the first human fluid in which MPs were identified [59], using pyrolysis combined with gas chromatography/mass spectrometry. This technique revealed that polymers such as PET, PS, and PE can translocate from the environment into the systemic circulation [60]. This foundational finding supported the hypothesis of systemic bioavailability of MPs, with important implications for child health given children’s physiological immaturity and heightened vulnerability.

More recently, MPs have also been detected in urine samples from children. A cross-sectional study conducted in Shenyang, China, examined 1000 primary school children aged 6 to 9 years, exploring the relationship between urinary MPs and behavioral outcomes [58]. MPs (PA, PP, PVC) were quantified using optical microscopy, and behavioral assessment was conducted via the Strengths and Difficulties Questionnaire. Higher urinary MP concentrations were significantly associated with increased emotional symptoms, conduct problems, hyperactivity, and peer relationship issues, alongside a reduction in prosocial behavior. No significant sex-related differences were found. These findings suggest that MP exposure may represent a potential neurodevelopmental risk in children. MPs have also been quantified in fecal samples, with detectable levels of PVC, PET, PE, and PA6 found in more than 85% of preschool children. This suggests a high level of oral exposure, likely influenced by behaviors such as bottle feeding and prolonged mealtimes, which may enhance plastic migration and ingestion.

Fecal analysis not only provides a non-invasive biomarker of oral exposure but also enables investigation of gut microbiota alterations [54].

Although more invasive, BALF has been used to detect inhaled MPs in children, particularly in urban settings, supporting the relevance of the respiratory route and highlighting the diagnostic potential of this matrix [36].

Saliva has recently been proposed as a non-invasive matrix for biomonitoring due to its direct contact with inhaled or ingested MPs; however, its application in pediatric settings remains limited.

Overall, these biological matrices offer complementary insights into different exposure routes and stages of MPs internalization, and their combined use could enhance early risk assessment in vulnerable populations such as children [37]. A recent study conducted in Kerman, Iran, monitored for the first time the presence of MPs in both saliva and on the hands of preschool children. The study included 100 children aged 3–6 years from five kindergartens across different districts. Following sample collection, digestion, and filtration, MPs were identified using optical microscopy and characterized by Micro-Raman spectroscopy. A total of 716 MPs were identified, 41.7% of which were black. After kindergarten attendance, MP levels increased by 55.9% in the hands and by 11.8% in saliva. Most particles measured under 100 μm, and chemical analysis revealed fibers composed primarily of polystyrene, nylon, and low-density polyethylene. The average number of handborne MPs rose from 1.85 to 4.2 post-entry, a statistically significant increase [37]. The study also found that environmental characteristics, such as the presence of tatami flooring and wall hangings, correlated with higher MP levels on surfaces. Given the absorbent nature of tatami, replacement with alternative materials was recommended, although further studies are needed to validate this finding. As kindergartens and play areas are increasingly utilized to support children’s cognitive and social development, attention to environmental exposures in these settings is essential for safeguarding child health [13].

## 7. Research Gaps and Limitations

Despite growing attention to the presence of microplastics in human biological matrices, critical gaps remain in understanding their accumulation in internal organs and long-term health effects in children. Three main areas require significant scientific and methodological advancement.

### 7.1. Detection Limitations Due to Instrumental Constraints

A major challenge in current research is the limited sensitivity of conventional techniques in detecting micro- and nanoplastics within human tissues. Standard optical or infrared microscopy may fail to identify particles smaller than 20 µm, which are the most likely to cross biological barriers and accumulate in internal organs [36]. Without the use of high-resolution tools such as scanning electron microscopy or Raman spectroscopy, studies may significantly underestimate actual exposure levels, particularly those relevant to pediatric health [61].

### 7.2. Lack of Standardized Sampling Protocols

The absence of standardized protocols for sample collection, processing, and analysis introduces considerable risk of contamination and false-positive findings. Variability in laboratory environments, storage conditions, and handling procedures undermines the reproducibility and comparability of results. This issue is especially critical in pediatric studies, where small sample volumes and strict ethical requirements further limit consistency. The establishment of harmonized methodologies is essential to reduce inter-study variability and enhance the validity of exposure assessments [62].

### 7.3. Lack of Information on All Organs in the Pediatric Age Group

To date, the scientific literature has reported the presence of microplastics in only a few pediatric tissues, specifically the lungs, thyroid gland, and intestinal microbiota. However, there is a complete absence of studies investigating microplastic accumulation in other essential organs during early life, such as the liver and kidneys. This is in sharp contrast to the adult population, for which the literature is increasingly abundant. An especially significant contribution is a recent prospective multicenter observational study published in The New England Journal of Medicine, which demonstrated the presence of MNPs, mainly polyethylene and polyvinyl chloride, within carotid atheromatous plaques. The study analyzed excised carotid plaque from 304 adult patients undergoing endarterectomy, detecting MNPs in over 58% of cases, with average concentrations of 21.7 μg/mg for PE and 5.2 μg/mg for PVC [63]. Importantly, the presence of MNPs in plaque was associated with a 4.5-fold increased risk of major cardiovascular events (myocardial infarction, stroke, or death) over a 34-month follow-up period. Electron microscopy confirmed that most plastic particles were <1 μm in size and were found both within macrophages and the amorphous matrix of plaques. Given these findings, it is implausible to assume that organs such as the kidney, which plays a central role in filtration and clearance, remain unaffected by microplastic accumulation, even during childhood. It is therefore of primary importance to raise awareness in the scientific community and to promote experimental studies specifically aimed at investigating the presence of microplastics in the organs and tissues of neonates and children.

### 7.4. Ethical and Practical Barriers to Tissue-Based Studies in Children

The lack of pediatric studies investigating MP accumulation in internal organs is largely due to ethical and practical constraints. While some evidence has been obtained from post-mortem or surgical specimens, the intentional collection of tissue samples for research purposes in children is not ethically permissible. As a result, data on tissue-specific distribution, organ-level toxicity, and long-term impact during development remain extremely limited. Alternative approaches, such as the analysis of clinically indicated surgical waste or the use of validated non-invasive biomarkers, may offer ethically acceptable pathways for advancing research in this area [64]. Taken together, these limitations highlight the urgent need for technological innovation, methodological standardization, and ethically responsible research strategies. Addressing these challenges is essential to advance our understanding of pediatric microplastic exposure and its potential health consequences. Given their increased physiological vulnerability and developmental sensitivity, children should be prioritized in future environmental health research and surveillance frameworks [36].

## 8. Conclusions

The exponential rise in environmental MNP contamination has raised growing concern regarding the implications of early-life exposure. Evidence increasingly points to widespread routes of pediatric exposure, via ingestion, inhalation, and dermal contact, linked to food, indoor dust, and direct contact with plastic materials. This scenario is particularly alarming given children’s unique metabolic features, developing organ systems, and prolonged lifetime exposure. Recent studies have suggested associations between urinary concentrations of MNPs and neurodevelopmental disorders, including emotional dysregulation, hyperactivity, and social impairments, as well as mitochondrial damage with downstream effects on hepatic, immune, and thyroid function [65]. Against this backdrop, prevention emerges as a cornerstone of pediatric protection. Reducing exposure from early infancy requires limiting the use of plastic products in direct contact with food, such as baby bottles, containers, and cutlery, and opting for clothing and furnishings made from natural fibers. Environmental design within educational and domestic settings also plays a critical role: recent studies have documented a higher concentration of MPs on children’s hands after time spent in environments with synthetic flooring materials such as tatami mats [37,66,67,68]. At a public health level, efforts must include educational campaigns directed at families and schools to promote awareness and behavior change. Parallel to this, the implementation of standardized, periodic monitoring of environmental and biological matrices (including urine, feces, and blood) is essential to evaluate exposure levels. Available data to date indicate that formula feeding, the use of plastic bottles for storing breast milk, single-use food packaging, viscose clothing, tatami mats in daycare centers, frequenting urban playgrounds made of plastic materials, parenteral nutrition, and, more generally, exposure to medical devices during hospital stays are all possible significant sources of MNP contamination in pediatric age. Reliable detection technologies, alongside pediatric biobanking and longitudinal observational studies, are urgently needed to define dose–response relationships, identify critical windows of susceptibility, and monitor long-term outcomes. Despite growing evidence, the absence of universally accepted protocols for internal microplastic monitoring and the scarcity of pediatric-specific data represent major limitations. Preliminary findings nonetheless suggest that MNP exposure in early life may contribute to inflammatory conditions, endocrine disruption, neurotoxicity, and even carcinogenic risk. These observations underscore the urgent need to regulate the presence of MNPs in products and foods intended for infants and children, particularly during the first 1000 days of life, a sensitive period when epigenetic programming and transgenerational health trajectories are shaped.

## 9. Future Perspectives

Future research must aim to fill the critical gaps in knowledge regarding MNP exposure and pediatric health, while also overcoming the ethical and technical limitations inherent in studying vulnerable populations. The development of sensitive and specific biomarkers, ideally detectable through non-invasive means, will be essential for monitoring internal exposure and establishing causal relationships. Advances in analytical techniques, such as Raman spectroscopy and high-resolution particle separation, may enhance the detection of nanoplastics, which are especially likely to penetrate biological barriers and accumulate in essential organs. Prospective, longitudinal pediatric cohorts integrating environmental, dietary, and clinical data are needed to delineate the cumulative effects of exposure, define dose–effect thresholds, and identify time windows of increased vulnerability. These efforts should be supported by international harmonization of sampling methods and detection protocols to ensure data comparability across settings. Healthcare professionals, especially pediatricians, neonatologists, and general practitioners, must be actively engaged in recognizing environmental plastic exposure as a relevant clinical factor. Their involvement will be key in translating scientific evidence into early guidance, risk stratification, and preventive care pathways. Moreover, incorporating environmental exposure screening into pediatric surveillance frameworks may help identify children at higher risk and inform targeted interventions. Finally, policymakers must be guided by emerging data to implement stricter regulations on the presence of MNPs in pediatric consumer products and foods. Protecting child health from plastic-related contaminants is not solely an environmental concern but a long-term investment in public health, with implications that extend across the lifespan and, possibly, generations.

## Figures and Tables

**Table 1:** State of the Art and Contribution of the Present Work.

What Was Already Known	What This Work Adds
Micro- and nanoplastics are ubiquitous environmental contaminants capable of bioaccumulating in human tissues, with potential toxicological effects. Most available knowledge derives from studies on adults or animal models, while data concerning the pediatric population are sporadic and fragmented.	This work represents the first narrative review entirely focused on the pediatric population. It provides a structured analysis of exposure routes specific to children (dietary intake, inhalation, dermal, and iatrogenic), describes the presence of MNPs in organs such as lungs, thyroid, and gut microbiota, and proposes possible non-invasive biomarkers. It offers an organized basis for the development of future clinical and experimental studies.

**Table 2:** Detected concentrations of microplastics in human adult tissues and biological fluids [45,47,48,49], modified. MPs = Microplastics.

Tissue/Fluid	MPs Concentration
Placenta	2.7–18 MPs/g
Endometrium	21 MPs/100 mg
Heart	0–7.75 MPs/g
Lungs	0.69–14.1 MPs/g
Kidneys	40.4 MPs/g
Liver	4.6 MPs/g
Spleen	1.1 MPs/g
Brain	4 800 µg/g
Colon	7.91 and 9.45 MPs/g
Endometrium	21 MPs/100 mg
Skin (hair, hands, and facial skin)	3.5; 2.1; 2.02 MPs per individual per day
Testis	11.6 MPs/g
Semen	0.23 MPs/g
Bronchoalveolar Lavage Fluid	9.18 ± 2.45 MPs/100 ml
Sputum	39.5 MPs/10 mL
Saliva	0.33 MPs per individual per day
Feces	1 to 50 MPs/g
Urine	0–9600 MPs/L
Blood	1.6 µg/ml
Breast Milk	0–2.72 Mps/g

## Data Availability

Data are publicly available on the reference sources we analyzed.
